# Lactate oxidation at the mitochondria: a lactate-malate-aspartate shuttle at work

**DOI:** 10.3389/fnins.2014.00366

**Published:** 2014-11-25

**Authors:** Daniel A. Kane

**Affiliations:** Department of Human Kinetics, St. Francis Xavier UniversityAntigonish, NS, Canada

**Keywords:** lactate, lactate dehydrogenases, mitochondria, pyruvates, malate aspartate

## Abstract

Lactate, the conjugate base of lactic acid occurring in aqueous biological fluids, has been derided as a “dead-end” waste product of anaerobic metabolism. Catalyzed by the near-equilibrium enzyme lactate dehydrogenase (LDH), the reduction of pyruvate to lactate is thought to serve to regenerate the NAD^+^ necessary for continued glycolytic flux. Reaction kinetics for LDH imply that lactate oxidation is rarely favored in the tissues of its own production. However, a substantial body of research directly contradicts any notion that LDH invariably operates unidirectionally *in vivo*. In the current Perspective, a model is forwarded in which the continuous formation and oxidation of lactate serves as a mitochondrial electron shuttle, whereby lactate generated in the cytosol of the cell is oxidized at the mitochondria of the same cell. From this perspective, an intracellular lactate shuttle operates much like the malate-aspartate shuttle (MAS); it is also proposed that the two shuttles are necessarily interconnected in a lactate-MAS. Among the requisite features of such a model, significant compartmentalization of LDH, much like the creatine kinase of the phosphocreatine shuttle, would facilitate net cellular lactate oxidation in a variety of cell types.

## Introduction: lactate dehydrogenase reaction

The reduction of pyruvate to lactate, catalyzed by lactate dehydrogenase (LDH; Pyruvate + NADH + H^+^ ⇌ Lactate + NAD^+^) in the cytosol of many cells, has been regarded as a metabolic “dead-end” (Luft, 2001; Quistorff and Grunnet, 2011a), or perhaps more aptly, a metabolic *cul de sac* (Barros, 2013), because lactate can only rejoin the metabolic network via pyruvate. In mammals, the LDH reaction is also considered to be “near-equilibrium” (Spriet et al., 2000; Quistorff and Grunnet, 2011a,b), meaning that the reaction is regulated chiefly by the concentrations of its reactants and products, rather than by more sophisticated means of allosteric regulation or covalent modification (Crabtree and Newsholme, 1978). Because the equilibrium for the LDH reaction lies far to the right (i.e., lactate formation favored) (Williamson et al., 1967), regardless of LDH isoform (Quistorff and Grunnet, 2011a,b), the implication might be that LDH rarely favors the reverse reaction (i.e., lactate oxidation) *in vivo*. Indeed, the mass action ratio ([lactate][NAD^+^]/[pyruvate][NADH][H^+^]) necessary for appreciable lactate oxidation would need to exceed the equilibrium constant for LDH. However, experimental evidence increasingly belies any notion that LDH operates unidirectionally *in vivo*, and supports that lactate serves as an important metabolic fuel for many tissues, including skeletal (Brooks et al., 1991; Bergman et al., 1999; Donovan and Pagliassotti, 2000) and cardiac muscle (Gertz et al., 1988; Chatham et al., 2001), liver (Skilleter and Kun, 1972; Kline et al., 1986), and brain (Schurr et al., 1988; Bouzier-Sore et al., 2006; Wyss et al., 2011; Funfschilling et al., 2012; reviewed in Barros, 2013). The purpose of the current Perspective is to forward a model in which lactate is central to the shuttling of energetic substrate between the cytosol (glycolysis) and the mitochondria (oxidative phosphorylation). Components of such a concept have been demonstrated in heart (Safer et al., 1971) and skeletal muscle (Schantz, 1986), were later expanded to a *lactate shuttle* perspective (Stainsby and Brooks, 1990; Brooks et al., 1999) and comprehensively reviewed (Gladden, 2004) and again commented upon (Gladden, 2007). The concept is particularly supported by recent research in neuronal cells (Gellerich et al., 2012, 2013; Rueda et al., 2014). While the concept outlined in the current Perspective is not new, *per se* (Safer et al., 1971), an apparent lack of conventional recognition or acceptance of its theoretical underpinnings, warrants further attention.

## The malate-aspartate shuttle

Due to the impermeability of the inner mitochondrial membrane to NAD^+^ and NADH, NADH generated by glycolysis under aerobic conditions depends on the indirect transfer of reducing equivalents into the mitochondria via the malate-aspartate shuttle (MAS) and glycerol-phosphate shuttle. These shuttles are also thought to regenerate cytosolic NAD^+^ necessary to support glycolytic flux at the NAD^+^-requiring glyceraldehyde 3-phosphate dehydrogenase reaction. The MAS has been demonstrated to be the predominant means by which this occurs in most oxidative tissues, and appears to constitute the principal NADH shuttle in mature neurons (Kauppinen et al., 1987; Ramos et al., 2003; Contreras and Satrustegui, 2009; Gellerich et al., 2012). It is also well established that during conditions of increased cellular energy demand and/or increased glycolytic flux (e.g., during strenuous exercise), as well as hypoxia, that the concentration of lactate will increase as the LDH reaction facilitates increased rates of cytosolic NAD^+^ regeneration (Robergs et al., 2004). In the brain, however, increasing the concentration of lactate in circulation (e.g., as during exercise) results in an increase in lactate disposal in the brain (Quistorff et al., 2008; van Hall et al., 2009; Boumezbeur et al., 2010; Dienel, 2012). It has also been suggested that the increased LDH activity (and, in turn, lactate production) simply compensates for the inability of the MAS to keep pace with cytosolic NAD^+^ demand (Schantz, 1986). In neurons, Ca^+2^ activation of the MAS and TCA cycle are competitive, such that lower levels of Ca^+2^ stimulates MAS activity by activating the glutamate/aspartate carrier (Contreras and Satrustegui, 2009), while higher concentrations of Ca^+2^ activate α-ketoglutarate dehydrogenase in the mitochondrial matrix, limiting the α-ketoglutarate available for the MAS (Contreras and Satrustegui, 2009). It is also possible that lactate is formed continuously in the cytosol, regardless of metabolic state, and that lactate oxidized at the mitochondria is coupled to the MAS. In isolated cardiac mitochondria, for example, the MAS exhibits an excess capacity, suggesting that the MAS activity alone is sufficient to maintain cytosolic NAD^+^ regeneration (Digerness and Reddy, 1976). Why, then, at rest and under fully aerobic conditions, would lactate be produced during glycolysis, if all the pyruvate *should* be going to the mitochondria for oxidative phosphorylation, and the MAS should be regenerating sufficient NAD^+^?

## Conventional (an)aerobic glycolysis

The appearance and disappearance of lactate during varying metabolic states has been a topic of much historical conjecture, controversy, and intrigue. There have been many reviews of the literature examining lactate metabolism, to which readers may be directed. Some of the more recent include (Cruz et al., 2012; Dienel, 2012; Kitaoka et al., 2012; Doherty and Cleveland, 2013; Newington et al., 2013; Brooks, 2014; Schurr, 2014; Todd, 2014). Unfortunately, many contemporary textbooks still use the metabolic fate of pyruvate to distinguish two types of glycolysis: aerobic (i.e., requiring oxygen) and anaerobic (i.e., without oxygen). In the presence of oxygen, it has been said, pyruvate will proceed to the mitochondria to meet its metabolic demise via oxidative phosphorylation, the net result of which is mitochondrial ATP resynthesis and oxygen consumption (i.e., respiration) (Voet et al., 2011). Conversely, when oxygen is limiting, the pyruvate is reduced to lactate in the cytosol by LDH, oxidizing its cofactor NADH in the process (Voet et al., 2011). A problem with this traditional construct is that it does not reconcile well with some recurring scientific observations. For example, it is well established that lactate is produced, and consumed, under fully aerobic conditions. Indeed, in healthy, normoxic individuals at rest in the postabsorptive state, it can be expected that approximately 50 μmol·min^−1^ of lactate are released from the brain alone (van Hall et al., 2009; van Hall, 2010). Clearly, lactate is more than a dead-end waste metabolite of anaerobic glycolysis; rather, shuttling of lactate throughout the organism provides useful perspective in which to interpret experimental observation.

## The lactate shuttle concept

Two lactate shuttle concepts have been forwarded which describe the movement and utilization of lactate within and between cells (Brooks, 1998). The *intra*cellular lactate shuttle hypothesis posits that lactate formed during glycolysis can be continuously used as an energy source within the same cell (Brooks, 1998). The *inter*cellular, or cell-cell lactate shuttle involves lactate generated and exported from a cell to be taken up and utilized by another cell (Brooks, 1998). The cell-cell lactate shuttle has gained general acceptance; the finer details of the intracellular lactate shuttle continue to be investigated, however. Recently, we demonstrated both a physical, as well as functional association of LDH with mitochondria in skeletal muscle (Elustondo et al., 2013). Using laser-scanning confocal microscopy, we confirmed the colocalization of LDH with mitochondrial membrane proteins in rat skeletal muscle. We found that mitochondria in saponin-permeabilized skeletal muscle fibers from rats oxidized lactate in the presence of NAD^+^, malate, and ADP (Elustondo et al., 2013); this was found similarly by another group in human fibers (Jacobs et al., 2013). The pyruvate was then transported into the mitochondria where it was further oxidized by pyruvate dehydrogenase (PDH), then the TCA cycle, with reducing equivalents stimulating respiration (Elustondo et al., 2013; Jacobs et al., 2013). We were able to inhibit respiration with just 5μM alpha-cyano-hydroxycinnamate, an inhibitor of mitochondrial pyruvate transport, further supporting that pyruvate, but not lactate is transported into the mitochondrial matrix. These findings support that LDH is strategically positioned to functionally interact with mitochondria, and suggest that lactate oxidation occurs near the outer surface of the inner mitochondrial membrane. How might an intracellular lactate shuttle operate in an intact cell? Let us return to the MAS.

**Figure 1:**
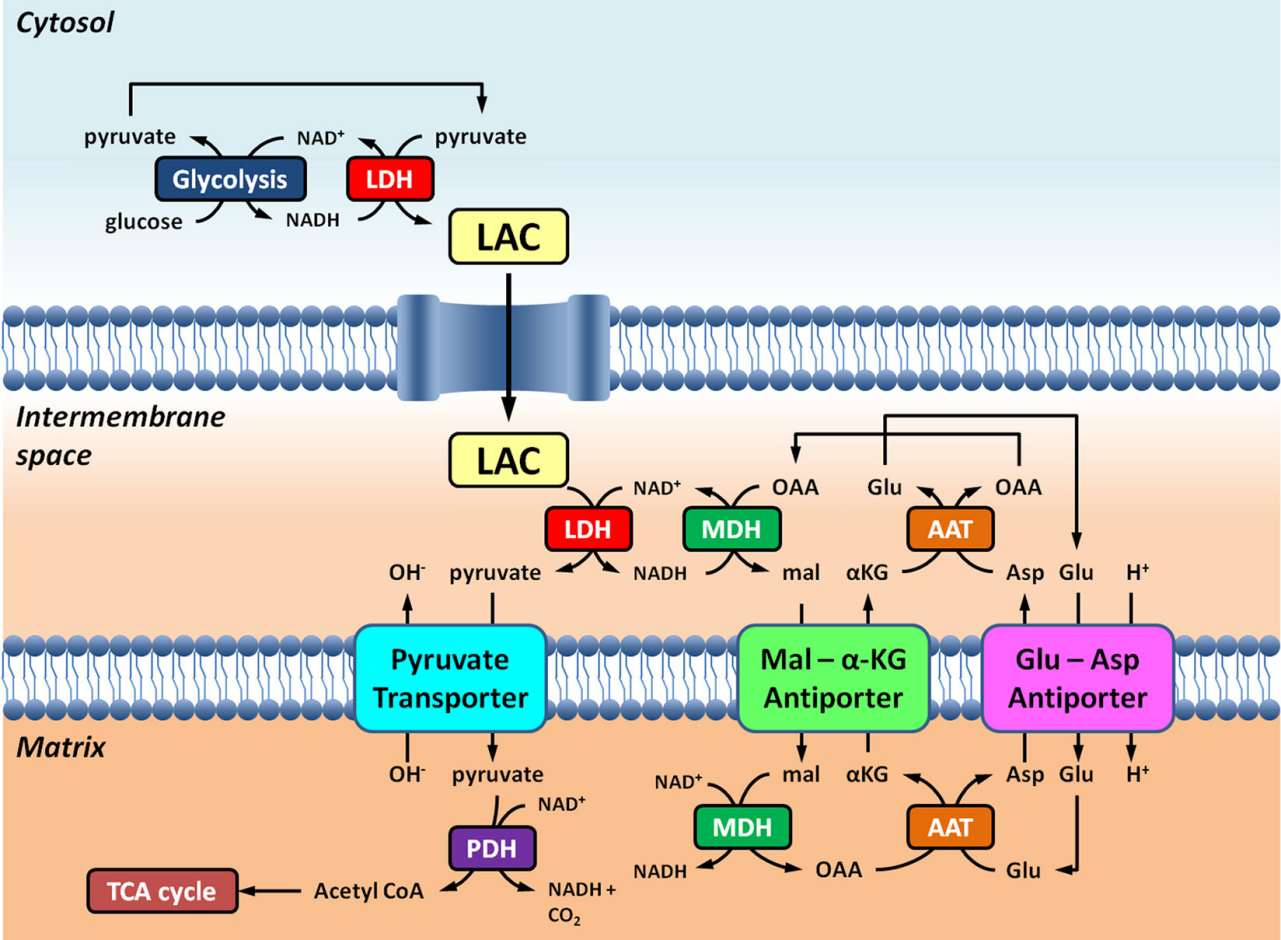
**Schematic representation of the link between glycolysis and lactate oxidation at the mitochondria outlined in this Perspective**. Overall, glycolysis yields pyruvate and NADH, in addition to ATP. NAD^+^ can be regenerated for glycolysis by the reduction of pyruvate to lactate (LAC) by lactate dehydrogenase (LDH). LAC can diffuse to the mitochondria where it is oxidized to pyruvate by LDH. NAD^+^ is regenerated by extra-matrix malate dehydrogenase (MDH) of the malate-aspartate shuttle. Pyruvate is subsequently transported across the inner mitochondrial membrane into the matrix, where it is then oxidized by pyruvate dehydrogenase (PDH) to acetyl CoA. Abbreviations: α-KG, alpha-ketoglutarate; Glu, glutamate; AAT, aspartate-aminotransferase; OAA, oxaloacetate; Mal, malate.

*In vivo*, cytosolic NAD^+^ could, in theory, be regenerated by malate dehydrogenase outside of the mitochondrial matrix, as part of the MAS. The literature gives some insight into different tissues and their mitochondrial shuttling activities. In the brain, the MAS has been considered the most important shuttle system for getting cytosolic NADH into the mitochondria (McKenna et al., 2006, and references therein); conversely, the glycerol-phosphate shuttle appears to be of lesser importance (Nguyen et al., 2003). Indeed, the intimate association of the MAS and the biosynthesis of neurotransmitter glutamate has been reported (Palaiologos et al., 1988). The published activities of the MAS measured in mitochondria isolated from rat brain are in the order of 26.7 nmol·min^−1^·mg^−1^ (Pardo et al., 2006). In synaptosomes, pharmacological inhibition of the MAS results in a pronounced (i.e. 50%) decrease in lactate oxidation (McKenna et al., 1993), supporting the model illustrated in Figure 1. Intracerebral production of lactate from ^13^C labeled glucose further supports the notion that lactate is an important fuel for neurons (Sampol et al., 2013).

It should be noted that the MAS may have its limits. At high cardiac workloads, it has been shown that the α-ketoglutarate/malate transporter of the inner mitochondrial membrane cannot compete with matrix α-ketoglutarate dehydrogenase for their shared substrate, α-ketoglutarate (O'Donnell et al., 2004). This results in a limiting effect to the MAS and its shuttling of NADH into the mitochondria. The net effect of this limitation to the MAS would be a rise in cytosolic lactate concentration as NAD^+^ regeneration via the LDH reaction would help to preserve homeostatic NAD^+^/NADH, even in the presence of adequate oxygen. Indeed, this is the classic phenomenon observed during especially strenuous exercise where lactate can accumulate in the blood, despite adequate oxygen availability.

## Intracellular compartmentalization of LDH: lessons from the phosphocreatine shuttle

The notion of shuttling compounds between the mitochondria and cytosol to meet the energetic demands of the cell using near-equilibrium enzymes is certainly not new. The phosphocreatine (PCr) shuttle involves distinct mitochondrial and cytosolic creatine kinase (CK) isoforms to essentially shuttle “high energy” phosphate from the mitochondria to the cytosol. Like the LDH reaction, the CK reaction (phosphocreatine + ADP + H^+^ ⇌ creatine + ATP), is considered to be “near-equilibrium,” favoring ATP resynthesis. However, experimental evidence demonstrates that in myocardial cells, only *cytosolic* CK is actually at, or near, equilibrium (Reviewed in Joubert et al., 2004). Mitochondrial CK, on the other hand, localized to the intermembrane space, is displaced from equilibrium, favoring net PCr resynthesis. By way of analogy, two distinct LDH populations are thought to be involved in the intracellular lactate shuttle: cytosolic and mitochondrial. The cytosolic LDH would be at or near equilibrium, whereas the mitochondrial LDH would be displaced from equilibrium. The cytosolic LDH would favor net lactate production, while the mitochondrial LDH would favor lactate oxidation. And much like the adenine nucleotide translocase (ANT), which transports ADP into the matrix across the inner mitochondrial membrane in exchange for ATP, facilitates the displacement from equilibrium for mitochondrial CK in the intermembrane space, so too would the pyruvate transporter continuously transport pyruvate, displacing the mitochondrial LDH reaction from equilibrium (Figure 1). Such a lactate shuttle would benefit from LDH localization in the intermembrane space near the inner mitochondrial membrane, bound to the outside of the outer mitochondrial membrane at contact sites of the outer and inner mitochondrial membrane, or both. If intracellular lactate oxidation is to occur at the mitochondria via compartmentalization, as with the PCr shuttle, the cellular localization of LDH in, at, or about the mitochondria would be a salient feature.

## Intercellular compartmentalization of lactate metabolism: the astrocyte-neuron lactate shuttle

A rich and growing body of neuroenergetic research also supports the existence of compartmentalized lactate metabolism among neighboring brain cells—namely, astrocytes and neurons (Reviewed in Pellerin and Magistretti, 2012). A variant of the intercellular lactate shuttle generally (Brooks, 2009), the astrocyte-neuron lactate shuttle (Pellerin and Magistretti, 2012) is based upon the idea that astrocytes are predominantly glycolytic, whereas neurons are more oxidative (Bouzier-Sore and Pellerin, 2013 and references therein). Using a metabolic modeling approach, it was argued that greater metabolic flux through PDH and the mitochondrial NADH shuttles in neurons compared to astrocytes necessarily dictates net lactate release by astrocytes and oxidation by neurons (Neves et al., 2012), supporting many experimental observations (reviewed in Bouzier-Sore and Pellerin, 2013). As mentioned previously, the MAS constitutes the major mitochondrial NADH shuttle in mature neurons (Kauppinen et al., 1987; Ramos et al., 2003; Contreras and Satrustegui, 2009; Gellerich et al., 2012). Hence, an important feature of the lactate-consuming neuron may well be its high MAS activity (Neves et al., 2012). Is it time for a *lactate-malate-aspartate shuttle*? Is there additional theoretical support for such a model in which lactate serves as a reducing equivalent?

## Regeneration of cytosolic NAD^+^

Lactate oxidation at the mitochondrion further makes sense of aerobic glycolysis by permitting cytosolic NAD^+^ regeneration by cytosolic LDH. Indeed, evidence in cultured cells points to a highly labile lactate/pyruvate ratio which varies to preserve homeostatic NAD^+^/NADH (Sun et al., 2012). This would be advantageous for the cell for a number of reasons. Firstly, it would provide an immediate means by which to regenerate NAD^+^ locally (i.e., in the cytosol, where glycolysis occurs); the greater relative diffusability of lactate (molecular weight = 89.07 g/mol) vs. NAD^+^ (molecular weight = 663.43 g/mol) means lactate can readily diffuse from the cell under conditions of increased glycolytic flux (e.g., intense exercise, hypoxic stress), while also being directed toward the mitochondria. During times of reduced cellular energy demand, continued lactate production during much lower rates of glycolytic flux would still be used to maintain homeostatic NAD^+^/NADH within the cell, as well as for continued coupling of intracellular lactate shuttling to the MAS.

## Proton shuttling and mitochondrial substrate transport

Lactate oxidation at the mitochondria makes sense of aerobic glycolysis because lactate production in the cytosol effectively consumes a proton (Robergs et al., 2004), which is thought to help mitigate the metabolic acidosis associated with increased ATP turnover and high rates of glycolysis (Robergs et al., 2004). The cytosolic concentration of lactate typically exceeds that of pyruvate by at least 10-fold, meaning that lactate, and not pyruvate is the predominant monocarboxylate entering the mitochondria intermembrane space (Brooks et al., 1999). By oxidizing lactate in the mitochondrial intermembrane space, protons would be released where they could contribute to the ΔpH component of the mitochondrial proton motive force across the inner membrane (Santo-Domingo and Demaurex, 2012), and/or be transported indirectly into the mitochondria by the MAS. As with the transport of inorganic phosphate and some other substrates and ions (Santo-Domingo and Demaurex, 2012), carrier-mediated transport of pyruvate across the inner mitochondrial membrane in rat liver mitochondria appears to be directly coupled to proton symport (or OH^−^ antiport) (Papa et al., 1971; Halestrap, 1975). Oxidation of lactate near the outer surface of the inner mitochondrial membrane, which releases a proton, would contribute to the ΔpH, and in turn, pyruvate transport into the matrix. Adjacent to the mitochondrial inner membrane, the LDH mass action ratio (i.e., concentrations of products/concentrations of reactions) could be largely facilitated by the “bleeding off” of pyruvate as it is continuously transported across the lactate-impermeable mitochondrial inner membrane, as well as a generous regeneration of NAD^+^ by the extra-matrix malate dehydrogenase of the MAS. In this model, the transport of pyruvate across in the inner mitochondrial membrane would directly influence the rate of lactate oxidation just outside the matrix. Lactate oxidation at the mitochondria would therefore be expected to be regulated indirectly at the PDH reaction in the matrix. This would be advantageous because unlike LDH, PDH is highly regulated via allostery and covalent modification. As mentioned, modeling predicts high PDH activity to dictate neuronal lactate consumption *in vivo* (Neves et al., 2012); and high PDH activity also characterizes lactate-consuming neurons in culture (Halim et al., 2010).

## Methodological considerations

If mitochondrial lactate oxidation is functionally linked to the activity of the MAS, then it would be important to include components of the MAS in *in vitro* analyses of mitochondrial lactate oxidation, such as malate itself or oxaloacetate. Malate is the likely choice, as it is routinely included to stimulate respiration *in vitro*, where it is transported into the matrix and oxidized by mitochondrial malate dehydrogenase to oxaloacetate. This oxaloacetate can then condense with acetyl coA formed, for example, when pyruvate is added. Including glutamate in addition to malate, allows full operation of the MAS at the level of mitochondrial respiration. An important, but sometimes overlooked aspect of appropriate mitochondrial lactate oxidation assessment is the inclusion of NAD^+^ as the requisite cofactor for the LDH reaction, and ADP as the phosphate acceptor to stimulate oxidative phosphorylation (i.e., state 3 respiration). Also, the extra-matrix component of the MAS involves the malate dehydrogenase reaction: oxaloacetate + NADH + H^+^ ⇌ malate + NAD^+^. Experimental protocols examining respiratory oxygen consumption in isolated mitochondria from muscle using high malate concentrations (e.g., 4 mM; Rasmussen et al., 2002), may favor the malate dehydrogenase reaction in the reverse direction (i.e., malate oxidation and NADH + H^+^ production) when added to the mitochondrial sample in combination with NAD^+^. Indeed, reversibility of the MAS has been observed in isolated hepatocytes (Berry, 1971) and mitochondria with reconstituted MAS (Kunz and Davis, 1991). The net effect of this MAS reversal on respiration would be to reduce malate from entering the mitochondria, forming oxaloacetate. More importantly, the reversal would prevent lactate oxidation to pyruvate, and subsequent transport and oxidation of the pyruvate in the matrix. Solutions to these methodological barriers to observing mitochondrial lactate oxidation *in vitro* involve including at least one component of the MAS. If adding malate, the appropriate concentration should be determined experimentally. Including ADP and NAD^+^ or NADH (recall, the MAS will generate NAD^+^ for the LDH reaction) is necessary also to observe appreciable mitochondrial lactate oxidation.

## Summary

A lactate-MAS is the interaction between the lactate and malate-aspartate shuttles to translocate reducing power to the mitochondria, particularly within oxidative, metabolically active cells.

### Conflict of interest statement

The author declares that the research was conducted in the absence of any commercial or financial relationships that could be construed as a potential conflict of interest.
